# Confinement Effect of Micro- and Mesoporous Materials on the Spectroscopy and Dynamics of a Stilbene Derivative Dye

**DOI:** 10.3390/ijms20061316

**Published:** 2019-03-15

**Authors:** Maria Rosaria di Nunzio, Ganchimeg Perenlei, Abderrazzak Douhal

**Affiliations:** 1 Departamento de Química Física, Facultad de Ciencias Ambientales y Bioquímica, and INAMOL, Universidad de Castilla-La Mancha, Avenida Carlos III, S/N, 45071 Toledo, Spain; mrosaria.dinunzio@uclm.es (M.R.d.N.); g.perenlei@qut.edu.au (G.P.); 2 Science and Engineering Faculty, Queensland University of Technology, Brisbane, QLD 4001, Australia

**Keywords:** H- and J-aggregates, host–guest interaction, push–pull molecule, porous silica-based materials, confinement effect

## Abstract

Micro- and mesoporous silica-based materials are a class of porous supports that can encapsulate different guest molecules. The formation of these hybrid complexes can be associated with significant alteration of the physico-chemical properties of the guests. Here, we report on a photodynamical study of a push–pull molecule, *trans*-4-(dicyanomethylene)-2-methyl-6-(4-dimethylaminostyryl)-4H-pyran (DCM), entrapped within faujasite-type zeolites (HY, NaX, and NaY) and MCM-41 in dichloromethane suspensions. The complex formation gives rise to caged monomers and H- and J-aggregates. Steady-state experiments show that the nanoconfinement provokes net blue shifts of both the absorption and emission spectra, which arise from preferential formation of H-aggregates concomitant with a distortion and/or protonation of the DCM structure. The photodynamics of the hybrid complexes are investigated by nano- to picosecond time-resolved emission experiments. The obtained fluorescence lifetimes are 65–99 ps and 350–400 ps for H- and J-aggregates, respectively, while those of monomers are 2.46–3.87 ns. Evidences for the presence of a charge-transfer (CT) process in trapped DCM molecules (monomers and/or aggregates) are observed. The obtained results are of interest in the interpretation of electron-transfer processes, twisting motions of analogues push–pull systems in confined media and understanding photocatalytic mechanisms using this type of host materials.

## 1. Introduction

Trapping organic molecules in the interior of meso/nanosized hybrid materials have been fascinating the scientific community since the 1980s for the possibility to form composites of great interest in advanced chemical and biological applications like catalysis, photonics, and drug delivery [1]. Among the large variety of porous supports, the most studied ones are those formed by the interaction of a guest molecule with silica-based materials like zeolites, MCM-41, SBA-15, and mesoporous silica nanoparticles. These constituents provide cavities and channels which allow to tune both the spectroscopy and dynamics of trapped molecules by affecting processes like proton-transfer [2,3,4], electron-transfer [5,6,7], and energy-transfer [8,9,10]. The variety of photophysical and photochemical events showed by these composites could be used to develop smart devices such as drug nanocarriers, nanosensors, nanoOLEDs, nanolasers, energy storage nanospace, and nanophotocatalysts [1]. Recently, we have reported on the photobehavior of (E)-2-(2-hydroxybenzyliden)amino-4-nitrophenol (HBA-4NP) trapped in zeolites and mesoporous materials [11,12]. The formed complexes contain monomers and aggregates of HBA-4NP enol forms which undergo an excited-state intramolecular proton-transfer reaction to originate keto-type phototautomers. The restriction to motion provided by the zeolite cavities causes a deceleration of the dynamics of the trapped keto species with respect to those observed in pure solution. Indeed, the initial dye concentration affects the strength of the guest–guest-interactions and, therefore, the time constants of the radiationless pathways. In particular, for the highest payload, both caged monomer and aggregates show the shortest emission lifetimes.

‘Push–pull’ systems, a class of molecules containing donor (D) and acceptor (A) moieties in their structure, undergo intramolecular charge-transfer (ICT) processes under light irradiation [13,14,15]. The excitation of the molecule usually leads at first to the S_1_-locally excited (LE) state whose electron density distribution and dipole moment values are similar to those of the ground-state, S_0_. In contrast, the CT state possesses a greater excited-state dipole moment. During an ICT process, the excited molecule can also suffer twisting motion. The ICT rate constant (10^−13^–10^−8^ s) thoroughly depends on the polarity of the medium. Numerous works have demonstrated the impact of the cavity/pore size of silica-based materials on the ICT reaction dynamics of the encapsulated guests [3,16,17,18,19,20,21,22,23,24].

**Scheme 1:**
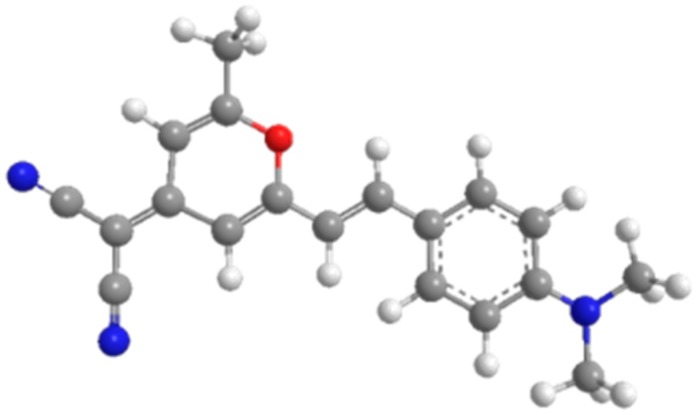
Molecular structure of DCM.

The molecular compound *trans*-4-dicyanomethylene-2-methyl-6-*p*-dimethylaminostyryl-4H-pyran (DCM, Scheme 1) is a typical push–pull chromophore. 

It is one of the most studied laser dyes due to its tunability over a wide range, high fluorescence efficiency, and stable photochemistry. DCM represents a promising candidate to be used in many areas and/or scientific applications and its complex photophysics deserves in depth investigations. The DCM structure, which is reminiscent of that of *trans*-stilbene, contains a dimethylamino and two cyanomethylene groups acting as the D and the A part, respectively. Because of a large charge separation upon excitation, the molecule shows larges solvent-polarity-dependent Stokes shifts and a huge change in dipole moment (20.7 D) from the ground- to the excited-state [25]. The LE-CT transition of DCM in solution has been widely investigated since the end of 1980s both experimentally and theoretically [26,27,28,29,30,31,32,33,34,35]. From these studies it has been concluded that, in contrast with other push–pull dyes, DCM never shows dual emission in solution. In particular, the CT state dynamics are detectable in polar solvent, being ICT in these cases the only significant deactivation process. On the other hand, in apolar solvents like cyclohexane the LE state is the unique fluorescent state with a lifetime of 16 ps [32]. The photobehavior of DCM has been also studied in confined media like micelles [36,37], lipid vesicles [38,39], microemulsion [40], human serum albumin (HSA) [41], polyvinyl carbazole (PVK) [42], and polypeptide-surfactant aggregate [43]. In these media, emission from the LE state has not been observed because the twisted intramolecular charge-transfer (TICT) transformation remains still ultrafast. The room temperature observation of DCM dual emission has been reported for the dye incorporated within the MCM-41 channels in the solid state [44]. Here, LE and CT states relax with lifetimes of 600 ps and 1.9 ns, respectively [44]. Very recently, we have reported another case of DCM dual emission for the dye trapped within a Zr-naphthalene dicarboxylic acid (Zr-NDC) metal organic framework (MOF) in diethyl ether suspensions [45]. For this system, the multi-exponential analysis of the fluorescence decays gives time constants of τ_1_ = 220 ps, τ_2_ = 790 ps, and τ_3_ = 2.5 ns. The shortest component τ_1_ is assigned to free DCM molecules, while τ_2_ and τ_3_ are due to the emission from DCM planar and TICT structures, respectively. In addition to electron-transfer, an energy-transfer process of 520 ps from Zr-NDC excimers to the included guests has been also observed in these experimental conditions.

Despite the large amount of reports on DCM in organized media, however, a more detailed study on the photobehavior of the dye entrapped in micro- and mesoporous materials is lacking. Here, we report on the photobehavior of DCM enclosed within faujasite-type zeolites (HY, NaX, and NaY) and MCM-41 using steady-state and pico- to nanosecond time-resolved emission spectroscopies. The DCM@host formation is confirmed by the spectral changes observed at both S_0_ and S_1_ levels. Different dye loadings are obtained depending on the properties of the used material. The restriction provided by the host provokes net blue shifts of both absorption and emission spectra due to the generation of several populations of H-type aggregates in addition to lesser amounts of J-aggregates and monomers. Moreover, trapped DCM may adopt a distorted structure because of the constraining environment with consequent loss of its planarity and π-conjugation. The nature of guest–guest and host–guest specific and non-specific interactions modifies the relative contribution of each population. The Stokes shift ($\Delta {\bar{v}}_{abs-em}^{max}$) values calculated for the formed complexes range from 4850 to 5850 cm^−1^. They are higher than the one observed in dichloromethane solution (4510 cm^−1^), thus suggesting that the trapped DCM molecules (monomers and/or aggregates) are still suffering the CT process. The lifetimes are 65–99 ps and 350–400 ps for H- and J-aggregates, respectively. They are shorter than those of monomers (2.46–3.87 ns) due to excitonic coupling. Increasing the initial concentration of the dye, these times become slightly shorter, also changing their relative contributions. These results may help for a better understanding of electron-transfer processes and twisting motions of related push–pull dyes in confined media.

## 2. Results and Discussion

### 2.1. Steady-State Observation

#### 2.1.1. Dichloromethane Solution

**Figure 1:**
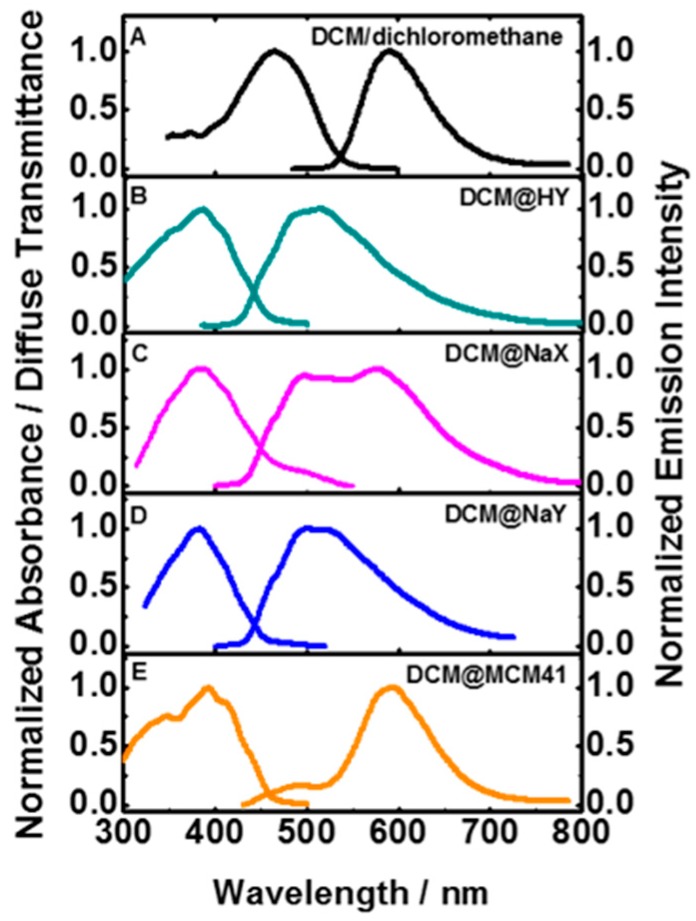
Normalized (to the maximum of intensity) UV-visible absorption/diffuse transmittance and fluorescence spectra of DCM in (**A**) dichloromethane solution and interacting with (**B**) HY, (**C**) NaX, (**D**) NaY zeolites, and (**E**) MCM-41 in dichloromethane suspensions. For all the composites, the initial dye concentration is 1 × 10^−4^ M. For emission, the excitation wavelengths are: 450 nm (for free DCM); 370 nm (for DCM@NaX and DCM@NaY); 380 (for DCM@HY); 340 nm (for DCM@MCM-41).

Figure 1A illustrates the normalized UV–visible absorption spectrum of DCM dissolved in dichloromethane. 

In pure solution, the dye exhibits a broad (full width at half maximum, fwhm, ~4600 cm^−1^), intense, and structureless band centered at 466 nm which is assigned to the S_1_ (π*) ← S_0_ (π) transition [35,46,47]. Additional high-energy absorption peaks located at 374 and 355 nm have been assigned to S_2_ ← S_0_ and S_3_ ← S_0_ transitions, respectively [35,46,47]. By the use of theoretical density functional theory (DFT) and complete active space self-consistent-field (CASSCF) approaches, it has been found that *trans* and *cis* DCM isomers possess comparable stabilities [47]. However, the most stable structure of the dye in the ground-state is the planar *trans*-form [47]. In these conditions, the thermal *trans* → *cis* isomerization does not occur as the energy barrier required for this process is relatively high. The emission band of DCM in pure solvent, originating from the S_0_ (π) ← S_1_ (π*) transition, shows its intensity maximum at around 590 nm (Figure 1A). Like absorption, it extends on a wide range (fwhm ~2300 cm^−1^) and it lacks in vibrational structure. The Stokes shift ($\Delta {\bar{v}}_{abs-em}^{max}$) of DCM in dichloromethane is 4510 cm^−1^, which is between that observed for the non-polar cyclohexane (3450 cm^−1^) [32] and methanol (5430 cm^−1^) [32]. These experimental facts evidence the CT nature of the emitting state of DCM in dichloromethane solutions.

#### 2.1.2. Complexes of DCM with HY, NaX, NaY Zeolites, and MCM-41

##### Diffuse Transmittance Spectra

The rapid change of the host’s color from white to yellow after its addition to a dichloromethane solution of DCM (1 × 10^−4^ M) proved the dye encapsulation and composite formation for all the investigated hybrid materials. Table 1 summarizes the calculated loading and entrapment efficiencies for DCM dye interacting with HY, NaX, NaY zeolites, and MCM-41 in our experimental conditions. 

The initial dye concentration is 1 × 10^−4^ M, while the quantity of used host material is 50 or 100 mg (for the zeolites or MCM-41, respectively). In the case of DCM@MCM-41 composite, the entrapment efficiency is referred to 50 mg of mesoporous material. Loading is an important parameter which gives information about the number of interacting molecules with the hosting framework. We obtained higher loading efficiencies for the three zeolites (6.3, 4.4, and 3.9 × 10^19^ DCM molecules/g_zeolite_ for HY, NaX, and NaY, respectively) with respect to MCM-41 (3.5 × 10^19^ DCM molecules/g_MCM-41_) due to a better affinity of DCM with the zeolite cages. The entrapment efficiency decreases, according to the loading, from 91 to 50% going from HY to MCM-41 (Table 1). In order to get more details on the dye distribution and the strength of the host–guest interaction, we rinsed three times the obtained composites with pure dichloromethane. The washing process removed additional dye molecules indicating that, during the complex formation, DCM may arrange inside (i.e., entrapped within the nano-cavities/channels) or outside (adsorbed on the external surface) the silica-based material. The interaction with the host is stronger for the inner molecules than for the outer ones, whose removal is promoted by simply washing the samples with the solvent. The different trapping abilities will be later discussed in this subsection considering the properties of the used materials. Figure 1B–E show the normalized diffuse transmittance (DT) spectra of DCM in presence of HY, NaX, NaY zeolites, and MCM-41 in dichloromethane suspensions starting from an initial dye concentration of 1 × 10^−4^ M. The DCM absorption spectrum changes to lower energies in presence of polar solvents. The polarity of MCM-41 lies among those of dichloromethane and 2-propanol [44], while that of zeolites are greater than methanol [1]. Therefore, we should expect a red-shift of the composite’s absorption spectra due to the more polar environment of the hosts. However, for all the cases, we observe net blue shifts (around 4450, 4580, 4720, and 4050 cm^−1^ for DCM interacting with HY, NaX, NaY, and MCM-41, respectively) of the absorption maxima with respect to the ones in dichloromethane solution. This suggests that the confinement effect, more than polarity, plays a major role in determining the spectroscopic behavior of the encapsulated dye. Confinement is strongly correlated with the molecular dimensions (host cavity diameter and guest size) and the nature of the host–guest interactions [1]. When DCM is accommodated inside the cavities/channels of the host materials, it may suffer a structural distortion with consequent loss of the molecular planarity. The shortest blue shift value calculated for the DCM@MCM-41 compound (4050 cm^−1^) is explained in terms of larger pore dimensions and, thus, less restriction (for the morphology of zeolites and MCM-41 materials, vide infra in this subsection). In presence of the three studied zeolites, DCM displays a new band with intensity maximum in the 382–386 nm range and additionally shoulders at around 350, 410, and 430 nm. On the other hand, with MCM-41 we observed a more vibrationally structured band, whose main peaks are located at 330, 351, 392 (maximum), and 415 nm and shoulders at 322, 371, and 434 nm. As suggested in Figure 1B–E, a small amount of free DCM exists in equilibrium with the encapsulated species, being more pronounced for NaX than for NaY; while for HY and MCM-41 it is almost imperceptible. This is in accordance with the different physical and chemical adsorption properties of the studied systems, which, in turn, affect the type of interactions with the dye.

##### Concentration Effect on the DT Spectra of the DCM@zeolite Complexes

**Figure 2:**
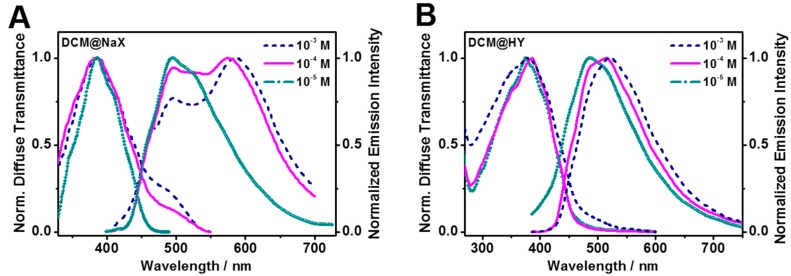
Normalized UV–visible diffuse transmittance and fluorescence spectra of DCM interacting with (**A**) NaX and (**B**) HY zeolites in dichloromethane suspensions at different initial dye concentrations: 1 × 10^−3^ (dashed line), 1 × 10^−4^ (solid line), and 1 × 10^−5^ (scattered-solid line) M.

To shed light on the origin of these absorption changes, we recorded additional DT spectra of DCM interacting with HY, NaX, and NaY zeolites using diluted (1 × 10^−5^ M) and concentrated (1 × 10^−3^ M) initial dye solutions. Figure 2 reports the results for the (A) DCM@NaX and (B) DCM@HY composites, while the spectral changes for DCM@NaY are reported in Figure S1 in the supplementary materials file. 

For emission, the excitation wavelength is 370 and 380 nm for DCM@NaX and DCM@HY, respectively. For NaX and NaY hosts, the effect of dye loading on both position and shape of the DT spectra was not as evident as for HY sample. The only remarkable difference stands in the free DCM absorption band, whose intensity increases with the initial dye concentration. We explain these discrepancies in terms of a HY stronger capability to encapsulate due to a larger affinity toward the guest. In presence of HY and increasing the initial dye concentration from 1 × 10^−5^ to 1 × 10^−4^ M, we observed a 10 nm-red shift of the main peak from 375 to 386 nm and a slight increase of the 410 nm band; while, for the dye-concentrated (1 × 10^−3^ M) composite, the absorption increases its intensity at both blue (~ 350 nm band) and red (~ 410 and ~430 nm bands) regions (Figure 2B). Moreover, a new absorption band appears at around 500 nm.

##### Deconvolution Analysis of the DT Spectra of DCM Interacting with the Studied Microporous and Mesoporous Materials

A spectral deconvolution (supposing a Gaussian shape of the absorption band) of the above-mentioned spectra is shown in Figure S2, while Table S1 collects the obtained results. At the lowest initial dye concentration (1 × 10^−5^ M), the deconvoluted DT spectrum consists of three bands centered at 360, 379, and 413 nm with % integral areas of 83, 6, and 11, respectively. As the dye loading increases ([DCM]_0_ = 1 × 10^−4^ M), both the 379 and 413 nm bands are redshifted to 414 and 434 nm and their intensities are reduced to 1 and 2% of the total area, respectively. On the other hand, the 360 nm band becomes broader and slightly blueshifted, with maximum at 353 and % area of 76, while a new component at 394 nm (% area = 21) nm is originated from the fit. At the highest used dye concentration (1 × 10^−3^ M), the 331 and 397 nm bands are the mayor components of the spectrum, with % areas of 63 and 36, correspondingly. A small contribution (1%) from the 505 nm-component was also observed from the fit. Based on these results, we suggest that, at the intermediate dye concentration (1 × 10^−4^ M), the 414 nm-band corresponds to the monomers population, whose contribution increases when diluting the starting solution ([DCM]_0_ = 1 × 10^−5^ M), while for the highest loading ([DCM]_0_ = 1 × 10^−3^ M) its signal is practically absent. The 353/394 and 434 nm components may correspond to H- and J-aggregates, respectively. The latter, together with monomers, were difficult to observe from the fit of the most concentrated sample, being probably masked by the 397 nm-component. Many organic planar chromophores are well known to aggregate even at low concentrations. The spatial restriction provided by the host encourages the establishment of specific (H-bonding donor/H-bonding acceptor, electrostatic) and non-specific (dipole–dipole, dipole-induced dipole) interactions between the adsorbed molecules, which may induce the formation of both H-(face-to-face) and J-(face-to-edge) aggregates [1,11,12,50,51,52,53,54,55,56]. The S_2_ ← S_0_ transition characterizing the H-aggregates absorption is located at higher energies, while the S_1_ ← S_0_ transition is spin forbidden. On the contrary, the absorption bands corresponding to the J-aggregates are narrow and located at lower energies and are related to S_1_ ← S_0_ transitions [56,57,58,59,60]. Taking into account the spectral positions and the effect of the initial dye concentration, we assign the band blueshifted by ~4200/1200 cm^−1^ (353/394 nm) with respect to that of the encapsulated monomers (414 nm) to H-aggregates and assign the band redshifted by ~1100 cm^−1^ (434 nm) to J-aggregates. These values are comparable to others reported for organic fluorophores in solution or adsorbed on crystalline surface [61,62,63]. Another possible assignment of the 353/394 nm bands may be a protonated form of DCM. It has been observed that protonation of DCM at the amine nitrogen atom by the strong Brönsted acid H_3_PW_12_O_40_ leads to a species whose absorption maxima are located at ~340 and 420 nm [35], which are quite similar to those obtained by the deconvolution analysis. However, it is difficult to apply a general rule to predict the nature of the absorption spectra of these complex systems. In addition, the relative spectral shifts of the aggregates, when present, are different for each specific case due to the intrinsic guest–guest and host–guest interactions as well as the nature of the surrounding solvent. Moreover, due to the heterogeneity of the samples, the resulting absorptions of trapped DCM becomes wider. This suggests that molecules may assume several positions and/or orientations with respect to the host system. As a last consideration, the 505 nm-component found with the fit of concentrated samples ([DCM]_0_ = 1 × 10^−3^ M) corresponds to free DCM in equilibrium with the encapsulated one. This result reflects the less encapsulation efficiency at higher dye concentrations ([DCM]_0_ ≥ 1 × 10^−3^ M). At lower concentrations ([DCM]_0_ = 1 × 10^−4^ M) the fit was unable to include the 505 nm-band due to its extremely low intensity. Based on these results, we propose the existence of DCM monomers along with H- and J-aggregates in the cases of NaX, NaY, and MCM-41 compounds, being the absorption spectra of these complexes very similar to the DCM@HY one. Even in this cases, protonation of the encapsulated guest should be considered. Indeed, high-level (λ < 360 nm) electronic transitions with vibrational structure are detectable in the DCM@MCM-41 spectrum indicating the formation of additional species, probably DCM isomers. Now, we will discuss the differences observed for the loading and organization/distribution of DCM in the used microporous and mesoporous materials. 

##### Differences in DCM Loading Capability of the Studied Microporous and Mesoporous Materials

To begin with, we outline the main properties of HY, NaX, and NaY in terms of morphology and chemical composition in order to understand the obtained DCM@zeolite loadings. The structure of X- and Y-type faujasite zeolites consists of a three-dimensional Si–O–Si and Si–O–Al interconnected network of spherical cavities (supercages) of ~11 Å diameter. Each supercage is then connected to four others through ~8 Å windows or pores. The microporous volume is equal to 0.34 and 0.29 cm^3^/g for NaY and NaX, respectively, which represents ~94 and 90% of the total porous volume for the two systems, in that order [49]. The Si/Al ratio is not constant for the two faujasites, being 1.24 for NaX and 2.86 for NaY. The presence of Al within the zeolite lattice is responsible for the formation of charge defects (the Si–O–Al-unit carries a negative charge) which generate new Brönsted sites, thus increasing the total acidity. The excess of negative charge affects both the number and position of the cunterions (Na^+^, in this case). The latter are known to occupy three different site positions: site I (16 cations per unit cell), on the hexagonal prism faces between the sodalite units; site II (32 cations per unit cell), located in the open hexagonal faces between the sodalite cage and the supercage; site III (38 cations per unit cell in the case of NaX and only 8 per unit cell in the case of NaY), on the walls of the supercage [64]. Both zeolite’s supercavity and pores sizes are comparable with the DCM dimensions (length = 16 Å, width = 7 Å), thus ensuring the molecule to enter and form aggregates. The latter may reside in the same or between neighboring supercages. As we said before, NaY displays a higher porosity than NaX. In addition to that, NaX zeolite has additional cations located in the α-cages (SIII) which reduce the available cage volume (overall capacity = 4.68 and 3.33 molec/cage for NaY and NaX, respectively) [49]. However, their loading capabilities toward DCM are, within the experimental error (15%), very similar (4.4 and 3.9 × 10^19^ DCM molecules/g_zeolite_ for NaX and NaY, respectively). On the other hand, the thermodynamic stability of the formed complex is higher for DCM@NaY than DCM@NaX according to the larger amount of desorbed DCM observed for NaX (Figure 1C) with respect to NaY (Figure 1D). These discrepancies can be elucidated considering the different electrostatic environments of the hosts. The lower Si/Al ratio of NaX (1.24) corresponds to 55% Si and 45% Al. This suggests that Al and Si T-atoms are alternatively located in the framework, generating a homogeneous electrostatic surrounding. On the contrary, in NaY the higher Si/Al ratio (2.86) makes the aluminum T-atoms more widely distributed, thus concentrating the charge more on the Al^+3^ centers. The more localized charge centers in NaY result in stronger host–guest interactions, and this explains the different stabilities observed for DCM@NaX and DCM@NaY complexes. The elimination of NH_3_ from NH_4_Y zeolite (the precursor of the studied HY, see Section 2) occurs in two endothermic steps: 1) desorption of NH_3_ from the small pores (sites I and I’) at 250 °C; 2) desorption of NH_3_ from the large pores (sites II) between 300 and 400 °C [65]. Dissociation of NH_3_ from NH_4_^+^ generates acidic sites within the zeolite framework due to the increased number of protons and, afterward, ≡Si-O(H^+^)-Al≡ species. The latter are probably formed by the combination of oxygen atoms from the zeolite lattice and the protons left after the NH_4_^+^ ions decomposition [66]. We expect that after 6 h of drying at 480 °C NH_3_ is fully liberated while protons are released within the framework. Therefore, HY presents, between the studied zeolites, the largest quantity of OH groups and the smallest amount of Na^+^ cations. The increased available free space (for the reduced number of Na^+^ cations) may be responsible for the highest loading ability (6.3 × 10^19^ DCM molecules/g_zeolite_) observed for HY. To continue with, we will compare the zeolite results with those obtained for DCM@MCM-41 materials. To do so, it is important to note that zeolites are crystalline materials with defined atom locations and pore sizes, while MCM-41 misses such crystallinity and order. They also possess different assemblies (hexagonal for MCM-41 and tetragonal for the zeolites) and sizes: pore/cavity diameters of 21–27 and ~11 Å for the mesoporous materials and the zeolites, respectively. Nevertheless, the main reason for the observed different loading capabilities stands in their composition: silanol groups for MCM-41 and Na^+^ cations, protons, and Si/Al atoms for the zeolites. Silanol groups are not as acidic as protons in zeolites. Moreover, MCM-41 does not contain Brönsted sites because there is no Al in its framework. As a consequence, the host–guest interactions should be more favorable for the zeolites (interactions with the Brönsted and Lewis acidic sites, Na^+^ cations, and aluminosilicate framework) than for MCM-41 (principally H-bonding interactions). These suggestions are supported by the lowest dye loading calculated for the MCM-41 host (3.5 × 10^19^ DCM molecules/g_MCM-41_). The latter is well known to be able to establish H-bonding with the encapsulated molecules thanks to the presence of the SiOH groups [18,19,67]. Indeed, a greater electron density is expected to be located at the cyano groups of the DCM molecule, thus generating a partial charge separation at its ground-state. This may induce the formation of dipole-dipole and/or dipole-induced dipole interactions between the guest and the interior walls of the used materials, thus helping the anchoring process.

##### Steady-State Emission Spectra

To study the photobehavior of the DCM@zeolite and DCM@MCM-41 complexes, we recorded their emission spectra upon excitation at 370/380 (for the DCM@zeolite complexes) and 340 nm (for DCM@MCM-41), where we mostly excite H-aggregates. Figure 1 shows the normalized emission spectra of the studied composites ((B) DCM@HY; (C) DCM@NaX; (D) DCM@NaY; (E) DCM@MCM-41) in dichloromethane suspensions (initial dye concentration = 1 × 10^−4^ M). The fluorescence spectra of the three dye@zeolite samples present a band with absorption features at 450 (shoulder), 487 (maximum), and 515 (maximum) nm or 457 (shoulder), ~500 (maximum), and ~520 (maximum) nm for HY or NaX and NaY zeolites, respectively. For the DCM@NaX composite, emission from the free dye (at around 576 nm) is also clearly visible from the spectrum. Remember that, in presence of NaX zeolite, DCM tends to dissociate easily with respect to HY and NaY cases (Figure 1C). When interacting with MCM-41, DCM exhibits a fluorescence spectrum with a shoulder at 453 nm and maxima at 486 and 500 nm together with an important contribution from the free form at around 590 nm. For all the investigated compounds, the fluorescence spectra are broader compared to the one recorded in pure solvent, with fwhm values of around 3910, 2870, 3380, and 2900 cm^−1^ for DCM@HY, DCM@NaX, DCM@NaY, and DCM@MCM-41, respectively (the fwhm in dichloromethane solution is ~2300 cm^−1^). This reflect the increased heterogeneity of the system, having a more complex ensemble of emitting fluorophores. The Stokes shift values observed for the DCM@zeolite complexes ($\Delta {\bar{v}}_{abs-em}^{max}$= 5415, 5590, and 5850 cm^−1^ for DCM@HY, DCM@NaX, and DCM@NaY, respectively) and for DCM@MCM-41 ($\Delta {\bar{v}}_{abs-em}^{max}$= 4850 cm^−1^) are higher than that observed in dichloromethane solution ($\Delta {\bar{v}}_{abs-em}^{max}$= 4510 cm^−1^), thus indicating that excited-state CT reactions may occur also for trapped DCM molecules (monomers and/or aggregates). The light absorption should induce conformational changes at the S_1_ level which may re-arrange the molecule in a planar conformation, thus encouraging the ICT process. The DCM confinement within HY, NaX, and NaY zeolites leads to a substantial decrease in the global fluorescence intensity via additional non-radiative decay channels like self-quenching processes. This has been also observed for several organic dye guests within such kind of host materials [1]. The fluorescence emission of DCM@MCM-41 is not so intense compared to that of the DCM@zeolite samples. In particular, it reduces when the excitation wavelength goes from 340 to 430 nm (Figure S3). The origin of the weaker fluorescence signal may reside in the less strong host–guest interactions between the dye and the MCM-41 materials which makes shorter the distance between two neighboring monomers. As a result, the electronic coupling in the dimer increases thus enhancing non-radiative deactivation channels. Another explanation to the reduced emission intensity of DCM@MCM-41 may be the establishment of non-radiative relaxation pathways through the OH groups of the host material.

##### Concentration Effect on the Emission Spectra of the DCM@zeolite Complexes

As suggested by Figure 2A and Figure S4, the emission behaviors of DCM interacting with NaX and NaY do not show a marked concentration effect. In particular, for the NaX system, emission from free DCM increases with the starting concentration of the dye (Figure 2A), in accordance with the absorption data. On the other hand, in presence of HY we observed a bathochromic shift of the emission when the dye concentration increases from 1 × 10^−5^ to 1 × 10^−3^ M (Figure 2B). This shift reflects the formation of more (H- and/or J-) aggregates together with an increase of the free DCM contribution to the total emission spectrum.

### 2.2. Time-Resolved Emission Measurements—Emission Decays of DCM Interacting with HY, NaX, and NaY Zeolites in Dichloromethane Suspensions 

In this section, we are going to describe the photodynamic properties of the formed complexes. To begin with, the emission decays of DCM in pure dichloromethane solution upon excitation at 371 nm and observing in the 525-650 nm range include two components with lifetimes of 0.2 (τ_1_) and 1.13 (τ_2_) ns (Figure S5 and Table S2). They correspond to the emission of different CT conformers formed after excitation of the dye. The shorter lifetime, τ_1_, contributes mostly in the blue part of the emission spectrum, while τ_2_ predominates at lower energies. Time-resolved transient absorption measurements have revealed a two-stage CT: the first step consists of charge separation and localization on the cyanomethylene acceptor groups, while the second one involves an out-of-plane twisting and pyramidalization of the dimethylamino group, which stabilizes the charge separation [33]. These results are also supported by theoretical calculations and isotopic substitution experiments [33].

#### 2.2.1. Concentration Effect on the Photobehavior of DCM@HY 

**Figure 3:**
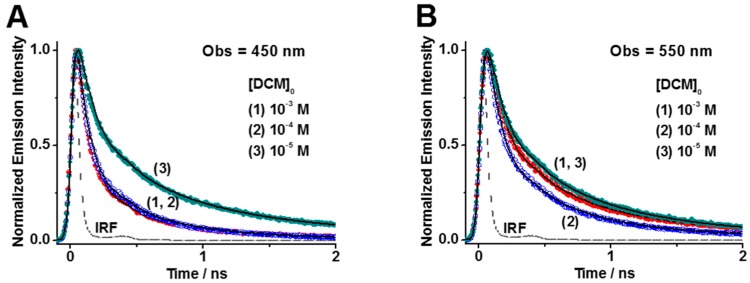
(**A**,**B**): Normalized (to the maximum of intensity) magic-angle emission decays of DCM@HY in dichloromethane suspensions upon excitation at 371 nm and observing at (**A**) 450 and (**B**) 550 nm at three different initial dye concentrations (1 × 10^−3^, 1 × 10^−4^, and 1 × 10^−5^ M). The solid lines are from the best multi-exponential fits to the experimental data. The instrument response function (IRF) is ~70 ps.

Figure 3 shows representative emission decays of DCM@HY composite in dichloromethane suspensions observing at (A) 450 and (B) 550 nm upon excitation at 371 nm at three different initial dye concentrations (1 × 10^−3^, 1 × 10^−4^, and 1 × 10^−5^ M). 

More observation wavelengths are reported in Figures S6 and S7 for DCM@HY complexes with [DCM]_0_ = 1 × 10^−3^ and 1 × 10^−4^ M, respectively. Table 2 gives the obtained time constants (τ_i_) and pre-exponential factors (a_i_) normalized to 100 after multi-exponential global fits. 

**Figure 4:**
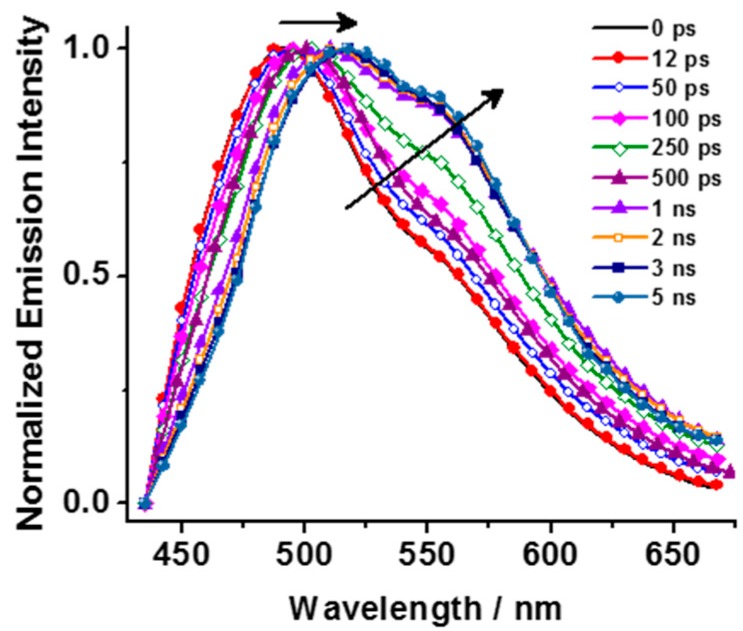
Normalized TRES of DCM interacting with HY zeolite prepared from a 1 × 10^−3^ M initial dye solution, gated at the indicated delay times after excitation at 371 nm.

It is worth noting that for all the studied samples the experimental decays were fit to three-exponential decay functions for observation wavelengths below 500 nm. Conversely, for lower energies we needed to add a fourth component due to the contribution of free DCM to the global signal. This additional component was fixed during the fit and corresponds to the average lifetime (τ_av_) of DCM in dichloromethane (1.10 ns). In agreement with the observed steady-state results, the obtained lifetime values and their corresponding fractional amplitudes depend on the loading. For diluted samples ([DCM]_0_ = 1 × 10^−5^ M), we observed decay times of 86 ps (τ_1_), 0.37 ns (τ_2_), and 3.88 ns (τ_3_) with related amplitudes (at 435 nm) of 73, 25, and 2%. The shortest component, τ_1_, contributes mainly at higher energies, while both τ_2_ and τ_3_ have more importance at the red part of the emission spectrum. Increasing the initial dye concentration, τ_1_ slightly decreases (τ_1_ = 79 and 75 ps for [DMC]_0_ = 10^−4^ and 10^−3^ M, respectively), while its fractional amplitudes become higher (a_1_% at 435 nm = 78 and 85 for [DMC]_0_ = 10^−4^ and 10^−3^ M, respectively). On the other hand, τ_2_ and τ_3_ preserve almost their initial values (τ_2_ = 0.36 and 0.35 ns; τ_3_ = 3.87 and 3.83 ns for [DMC]_0_ = 10^−4^ and 10^−3^ M, respectively), with contributions which are decreasing for both components. Based on the excitonic theory, the H-aggregates lifetime should be shorter than the J-aggregates lifetime because of their forbidden transition. Therefore, we assign the longest lifetime, τ_3_, to the monomers (neutral or even protonated), while τ_1_ and τ_2_ correspond to the lifetimes of the H- and J-aggregates, respectively. It is important to note that, observing at 450 nm (Figure 3A), the emission decays trend is consistent with the data collected in Table 2. However, at 550 nm (Figure 3B), where emission from free DCM also contributes to the global signal, the tendency is not as systematic as the one at 450 nm. In order to get deeper insight on the photodynamics of the DCM@HY composites, we recorded time-resolved emission spectra (TRES) using concentrated (1 × 10^−3^ M) and intermediate (1× 10^−4^ M) dye initial solutions (Figure 4 and Figure S8). 

For the most concentrated sample, we can differentiate three bands in the TRES centred at 491, 518, and 555 nm. The long-living band is assigned to the emission of H-aggregates, while those having the maximum at 518 and 555 nm belong to J-aggregates and neutral/protonated monomers, respectively, in accordance with the steady-state results. We observed similar dynamics when the starting dye concentration decreases to 1 × 10^−4^ M (Figure S8).

#### 2.2.2. Photobehavior of DCM within HY, NaX, and NaY Zeolites

**Figure 5:**
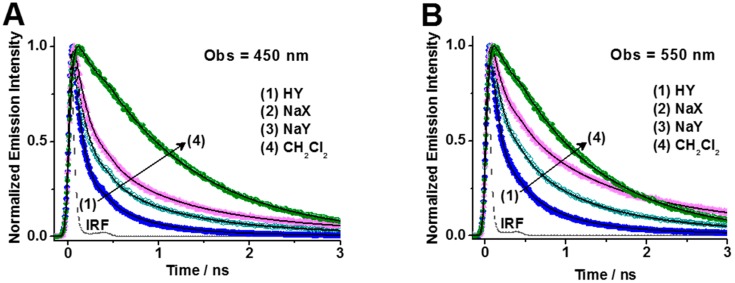
Normalized (to the maximum of intensity) magic-angle emission decays of DCM in presence of HY, NaX, and NaY zeolites (initial dye concentration: 1 × 10^−4^ M) in dichloromethane suspensions and comparison with the free dye in dichloromethane solution upon excitation at 371 nm and observing at (**A**) 450 or (**B**) 550 nm for the three complexes. The observation wavelength for the free dye is, in both cases, 600 nm. The solid lines are from the best multi-exponential fits to the experimental data. IRF is ~70 ps.

The nature of the host surrounding plays a key role in determining the fate of caged fluorescent probes. Figure 5 shows the emission decays of DCM in pure dichloromethane solution and in presence of HY, NaX, and NaY zeolites (initial dye concentration: 1 × 10^−4^ M) in dichloromethane suspensions excited at 371 nm and collected at 450 nm (Figure 5A) and 550 nm (Figure 5B). 

More observation wavelengths for the DCM@NaX and DCM@NaY composites are given in Figures S9 and S10. For all the studied composites, the fits give three components in addition to a fourth one corresponding to free DCM which was, also in these cases, kept fixed to 1.10 ns. Table 3 only reports the values of the emission lifetimes and pre-exponential factors for λ < 500 nm, where no emission from free dye is observed. The whole investigated range is reported in Table S3. 

**Scheme 2:**
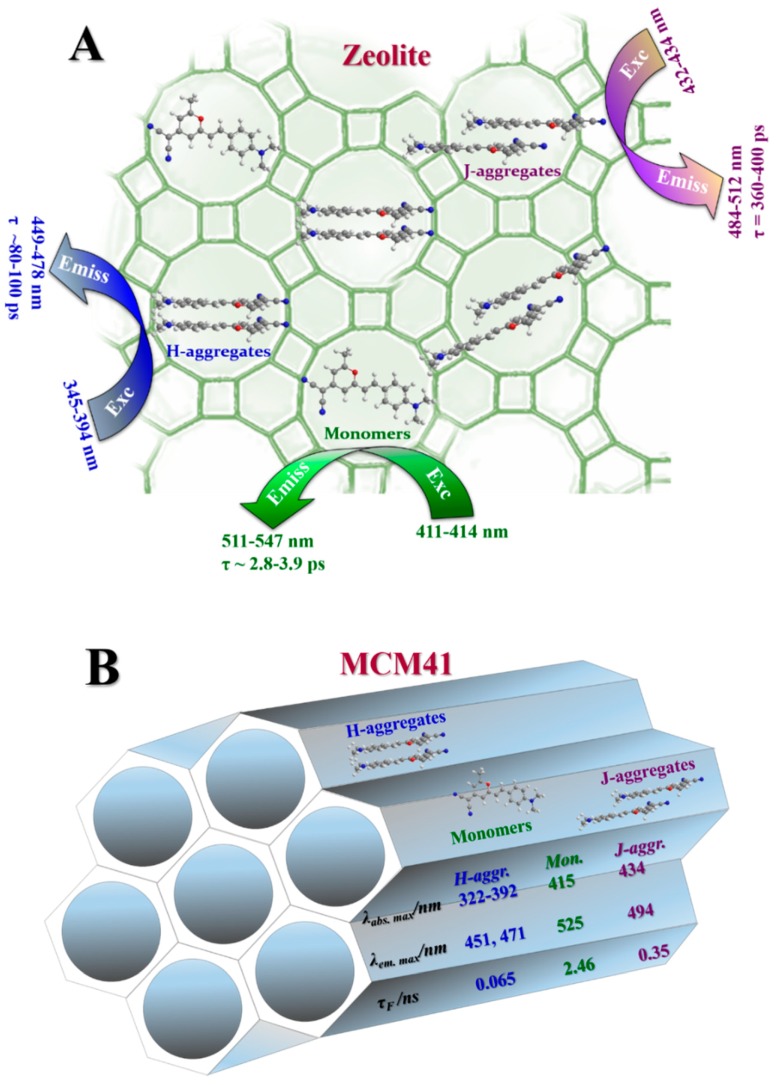
Values of the wavelengths of absorption and emission maxima together with the fluorescence lifetimes of DCM confined within the studied (**A**) zeolites (HY, NaX, and NaY) and (**B**) MCM-41 in dichloromethane suspensions.

The emission decays become clearly longer going from HY to NaX, NaY, and the pure solvent. We found time constants with values of τ_1_ = 96 and 99 ps, τ_2_ = 0.40 and 0.36 ns, and τ_3_ = 2.75 and 3.30 ns for DCM interacting with NaX and NaY, respectively. We assign these components to H- and J-aggregates and neutral/protonated monomers in a similar way to the HY case. While J-aggregates show comparable lifetime values, the dynamics of H-aggregates become shorter going from NaY to HY due to the highest loading found for the latter. Larger loadings make shorter the intermolecular distances, thus favoring non-radiative channels. The lifetime of monomers is reduced going from HY (3.87 ns) to NaY (3.30 ns); this suggests a higher monomer concentration in NaY, which could favor self-quenching processes. The shortest value found for NaX (2.75 ns) can be explained by additional interaction of the monomers with Lewis acidic sites or with the Na^+^ ions (electrostatic interactions). The contribution of H-aggregates increases at shorter wavelengths and it is the highest for DCM@HY, being 78, 70, and 65% for HY, NaX, and NaY zeolites, respectively. In the case of J-aggregates, the maxima pre-exponential values are in the 475–500 nm region. We did not observe appreciable changes in the J-aggregate contribution within the studied hosts probably because these values are altered by the presence of the free dye, whose lifetime is quite close to the J-aggregates one. Finally, monomers are more abundant at lower energies, with contributions which are the highest ones for NaY and then decrease going from HY to NaX, in accord with the steady-state results. A summary of the spectral and dynamical properties of DCM encapsulated onto the studied zeolites is shown in Scheme 2A.

#### 2.2.3. Emission Decays of DCM Interacting with MCM-41 in Dichloromethane Suspensions.

**Figure 6:**
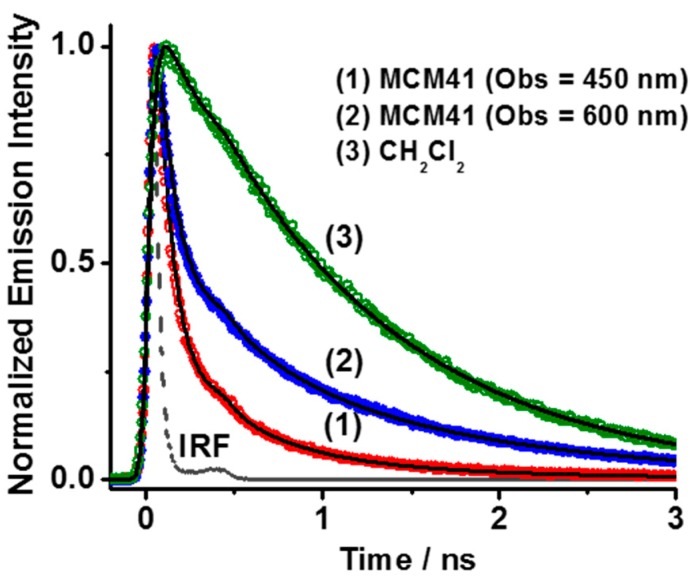
Normalized (to the maximum of intensity) magic-angle emission decays of DCM@MCM-41 composite (initial dye concentration: 1 × 10^−4^ M) in dichloromethane suspensions and comparison with the free dye in dichloromethane solution exciting at 371 nm and observing at (**1**) 450 nm and (**2**) 600 nm. The observation wavelength for the free dye is 600 nm. The solid lines are from the best multi-exponential fits to the experimental data. IRF is ~70 ps.

Figure 6 displays the emission decays of DCM in dichloromethane solution and in contact with MCM-41 (initial dye concentration: 1 × 10^−4^ M) in dichloromethane suspension exciting at 371 nm and observing at 450 and 600 nm.

Results of experiments gating at more observation wavelengths are shown in Figure S11, Table 3 and Table S3 contain the information on the dynamics of this composite. When interacting with MCM-41, DCM shows the shortest time constants as we were expecting based on the steady-state results. The obtained lifetimes are, in fact, 65 ps, 0.35 ns, and 2.46 ns for H-aggregates, J-aggregates, and neutral/protonated monomers, respectively. In a similar way to the previously analyzed systems, H-aggregates are the dominant species at higher energies, while monomers prevail on the reddest side of the emission spectrum. J-aggregates have a maximum contribution around 500 nm. Scheme 2B resumes the behavior of DCM@MCM-41 complex at its ground- and excited-states.

## 3. Materials and Methods

DCM, anhydrous dichloromethane (spectroscopic grade ≥99.8%), NaX, NaY, ammonium NaY, and MCM-41 were acquired from Sigma-Aldrich (Madrid, Spain). Steady-state UV–visible absorption and DT spectra were performed with the use of a Jasco V-670 double-beam spectrophotometer furnished with a 60-mm integrating sphere (ISN-723). Emission spectra were performed with a Fluoromax-4 (Jobin-Yvone) (Paris, France). Fluorescence decays were recorded with a picosecond time-correlated single-photon-counting (TCSPC) spectrophotometer (FluoTime 200, PicoQuant, Berlin, Germany) [68]. The emission intensity, gated at the magic angle of 54.7°, was perpendicular to the excitation beam and it was checked at distinct wavelengths. We excited the samples using a 40-ps pulsed diode laser focused at 371 nm (< 5 mW, 40 MHz repetition rate). The IRF (~ 70 ps) was measured by a standard LUDOX (Sigma-Aldrich) solution (optical path length = 1 cm). The resulting decay data were evaluated by the FluoFit software set of PicoQuant (Berlin, Germany). Exponential decay functions were convoluted to IRF to fit the experimental decays. The shorter component determined after a convolution process show a time constant of 15 ps. The number of exponentials were cautiously selected based on the reduced χ^2^ values (always ≤ 1.1) and the residuals distributions. The calculated time constant errors were below 20%. The laboratory temperature was 293 K. DCM@zeolite and DCM@MCM-41 composites were prepared by adding 50/100 mg of dried zeolite/MCM-41 (6 h at 480 °C and then 2 h within the oven turned off) to 10 mL of dichloromethane containing different concentrations (1 × 10^−5^–1× 10^−3^ M) of DCM and stirring the solutions at room temperature overnight. Afterward, the composites were washed three times (10 min, 4200 rpm) with pure dichloromethane and dried under vacuum at room temperature. The DCM loading, expressed as number of DCM molecules per g of host material, was calculated as

$$\frac{\left[\left(A_{i}-A_{s}\right)/\varepsilon \cdot l\right]\times V\times N_{A}}{g_{host}}$$

In the above Equation, A_i_ and A_s_ are the absorbance values of DCM at its intensity maximum in the initial and supernatant solution, respectively; ε is the molar extinction coefficient of DCM; *l* is the optical path length; V is the volume of the solution; N_A_ is the Avogadro’s number. The entrapment efficiency is given by ((A_i_ − A_s_)/A_i_) × 100. The estimated uncertainty of the DCM loading was ~15%. All the suspensions were prepared adding 3 mg of a dried material to 3 mL of dichloromethane solution. In order to avoid the absorption of moisture from the atmosphere, the total time for the sample preparation was less than 2 min.

## 4. Conclusions

The present work deals with the spectroscopic and photodynamical behavior of the push–pull DCM dye trapped within faujasite-type zeolites (HY, NaX, and NaY) and MCM-41. The spatial arrangement and chemical composition of the used host material affect the entrapment efficiency, the latter being higher for the three zeolites (6.3, 4.4, and 3.9 × 10^19^ DCM molecules/g_zeolite_ for HY, NaX, and NaY, respectively) with respect to MCM-41 (3.5 × 10^19^ DCM molecules/g_MCM-41MCM-41_) due to stronger interactions of DCM with the zeolite frameworks. The interaction between DCM molecules and the mesoporous hosts induces remarkable changes at both S_0_ and S_1_ states of the encapsulated dye. For instance, a blue shift in both the absorption and emission spectra was observed when DCM is trapped within the hosts, and this phenomenon was attributed to a preferential formation of H-aggregates with respect to J-aggregates and monomers, along with a probable distortion of the molecular structure. The relative contribution of the different populations (neutral/protonated monomers and H-/J-aggregates) is influenced by the type of interaction. Moreover, an increase in the Stokes shift values was observed for trapped DCM (ranging to 4850 to 5850 cm^−1^) in comparison to the one obtained in dichloromethane solution (4510 cm^−1^), reflecting the emission of CT species (monomers and/or aggregates) of encapsulated DCM molecules. Time-resolved experiments elucidate the photodynamics behavior of trapped DCM, showing a multiexponential behavior with time constants of τ_1_ = 65–99 ps, τ_2_ = 350–400 ps, and τ_3_ = 2.46–3.87 ns assigned to the emission lifetime of H-aggregates, J-aggregates, and monomers, respectively. The shorter values of H- and J-aggregates compared to that of monomers reflects the strong electronic coupling of the two interacting monomers. Furthermore, by increasing the dye concentration we observed a shortening of the lifetimes together with changes in their relative contributions, corroborating our assignment. In this report we present, for the first time, the formation of caged H- and J-type aggregates of DCM within microporous and mesoporous materials. These results open a via for the analysis of electron-transfer processes and twisting motions of related push–pull dyes in confined media, leading to a better understanding of their possible application in the field of nanocatalysis.

## Figures and Tables

**Table 1:** Values of cavity/pore diameter and total pore volume of the used zeolites (HY, NaX, and NaY) and MCM-41 together with the loadings and entrapment efficiencies for DCM dye interacting with the studied systems.

Composite	Cavity/Pore diameter (Å)	Total Pore Volume (cm^3^/g)	DCM_loading_ (10^19^ DCM molecules/g_host_)	Entrapment Efficiency (%)
DCM@HY	~ 11 ^a^	0.33 ^b^	6.3	91
DCM@NaX	~ 11 ^a^	0.29 ^c^	4.4	63
DCM@NaY	~ 11 ^a^	0.34 ^c^	3.9	57
DCM@MCM-41	21–27 ^d^	0.98 ^d^	3.5	50

^a^ From IZA database; ^b^ from [48]; ^c^ from [49]; ^d^ from the property description provided by the company.

**Table 2:** Values of time constants (τ_i_) and normalized (to 100) pre-exponential factors (a_i_) obtained from the fit of the emission decays of DCM interacting with HY prepared from DCM solutions of 1 × 10^−3^, 1 × 10^−4^, and 1 × 10^−5^ M upon excitation at 371 nm and observing at 435 and 450 nm. The estimated uncertainty of the time constants, considering the errors from the experiments as well as those arising from the multi-exponential fit of the signals, was between 13 and 20%.

DCM@HY		H-aggregates		J-aggregates		Monomers		Free DCM	
[DCM]_0_/M	λ_em_/nm	τ_1_ (± 15)/ps	a_1_/%	τ_2_ (± 0.07)/ns	a_2_/%	τ_3_ (± 0.58)/ns	a_3_/%	τ_4_ (± 0.16)/ns	a_4_/%
10^−3^	435	75	85	0.35	14	3.83	1	1.10	−
	450		82		17		1		−
	500		62		20		2		16
	550		57		19		3		21
	625		55		15		5		25
10^−4^	435	79	78	0.36	20	3.87	2	1.10	−
	450		75		22		3		−
	500		62		26		3		9
	550		57		23		9		11
	625		53		20		10		17
10^−5^	435	86	73	0.37	25	3.88	2	1.10	−
	450		68		28		4		−
	500		51		35		8		6
	550		43		34		15		8
	625		34		33		20		13

**Table 3:** Values of time constants (τ_i_) and normalized (to 100) pre-exponential factors (a_i_) obtained from the fit of the emission decays of DCM interacting with HY, NaX, NaY, and MCM-41 prepared from DCM solutions of 1 × 10^−4^ M upon excitation at 371 nm at the observation wavelengths indicated in the Table. The estimated uncertainty of the time constants, considering the errors from the experiments as well as those arising from the multi-exponential fit of the signals, was between 15% and 20%.

DCM@host		H-aggregates		J-aggregates		Monomers	
Host	λ_em_/nm	τ_1_ (± 15)/ps	a_1_/%	τ_2_ (± 0.07)/ns	a_2_/%	τ_3_ (± 0.58)/ns	a_3_/%
HY	435	79	78	0.36	20	3.87	2
	450		75		22		3
NaX	435	96	70	0.40	29	2.75	1
	450		68		31		1
	475		65		34		1
NaY	435	99	65	0.36	31	3.30	4
	450		59		33		8
	475		55		33		12
MCM-41	450	65	86	0.35	13	2.46	1
	475		73		24		3

## Supplementary Materials

Supplementary materials can be found at https://www.mdpi.com/1422-0067/20/6/1316/s1.

## Abbreviations

DCM	*trans*-4-(dicyanomethylene)-2-methyl-6-(4-dimethylaminostyryl)-4H-pyran
CT	charge-transfer
HBA-4NP	E)-2-(2-hydroxybenzyliden)amino-4-nitrophenol
D	donor
A	acceptor
ICT	intramolecular charge-transfer
LE	locally excited
HSA	human serum albumin
PVK	polyvinyl carbazole
TICT	twisted intramolecular charge-transfer
Zr-NDC	Zr-naphthalene dicarboxylic acid
MOF	metal organic framework
DFT	density functional theory
CASSCF	complete active space self-consistent-field
DT	diffuse transmittance
τ_i_	time constant
a_i_	pre-exponential factor
τ_av_	average lifetime
TRES	time-resolved emission spectra
TCSPC	time-correlated single-photon-counting
IRF	instrument response function
A_i_	initial absorbance
A_s_	supernatant absorbance
ε	molar extinction coefficient
*l*	optical path length
V	volume
N_A_	Avogadro’s number
