# Supplementary material for: Confinement Effect of Micro- and Mesoporous Materials on the Spectroscopy and Dynamics of a Stilbene Derivative Dye
Source: Int J Mol Sci. 2019 Mar 15;20(6):1316. doi: 10.3390/ijms20061316 (PMC6471191; doi:10.3390/ijms20061316)
Supplement: Supplementary file 1 [file ijms-20-01316-s001.pdf]

International Journal of *Molecular Sciences*

Confinement Effect of Micro- and Mesoporous Materials on the Spectroscopy and Dynamics of a Stilbene Derivative Dye

Maria Rosaria di Nunzio [1], Ganchimeg Perenlei [1,2] and Abderrazzak Douhal [1,*]

[1] Departamento de Química Física, Facultad de Ciencias Ambientales y Bioquímica, and INAMOL, Universidad de Castilla-La Mancha, Avenida Carlos III, S/N, 45071 Toledo, Spain; mrosaria.dinunzio@uclm.es (M.R.d.N.); gana_d3@yahoo.com (G.P.)

[2] On leave from Science and Engineering Faculty, Queensland University of Technology, Brisbane, QLD 4001, Australia

* Correspondence: abderrazzak.douhal@uclm.es (A.D.); Tel.: +34-925-265717

Supplementary Materials

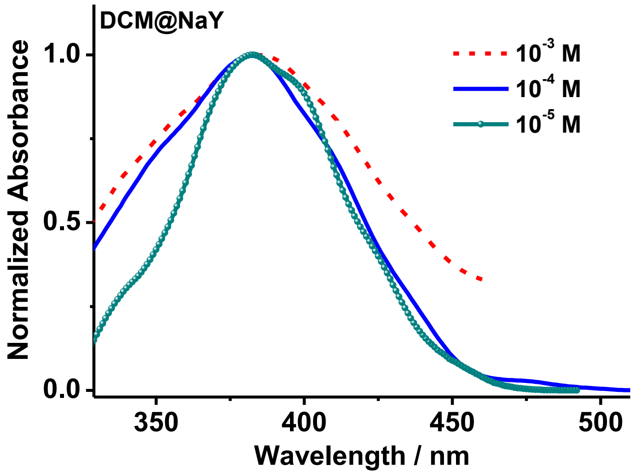

Figure S1. Normalized (to the maximum of intensity) UV-visible diffuse transmittance spectra of DCM interacting with NaY zeolite in dichloromethane suspensions at different initial dye concentrations: 1 × 10^{-3} (dashed line), 1 × 10^{-4} (solid line), and 1 × 10^{-5} (scattered-solid line) M.

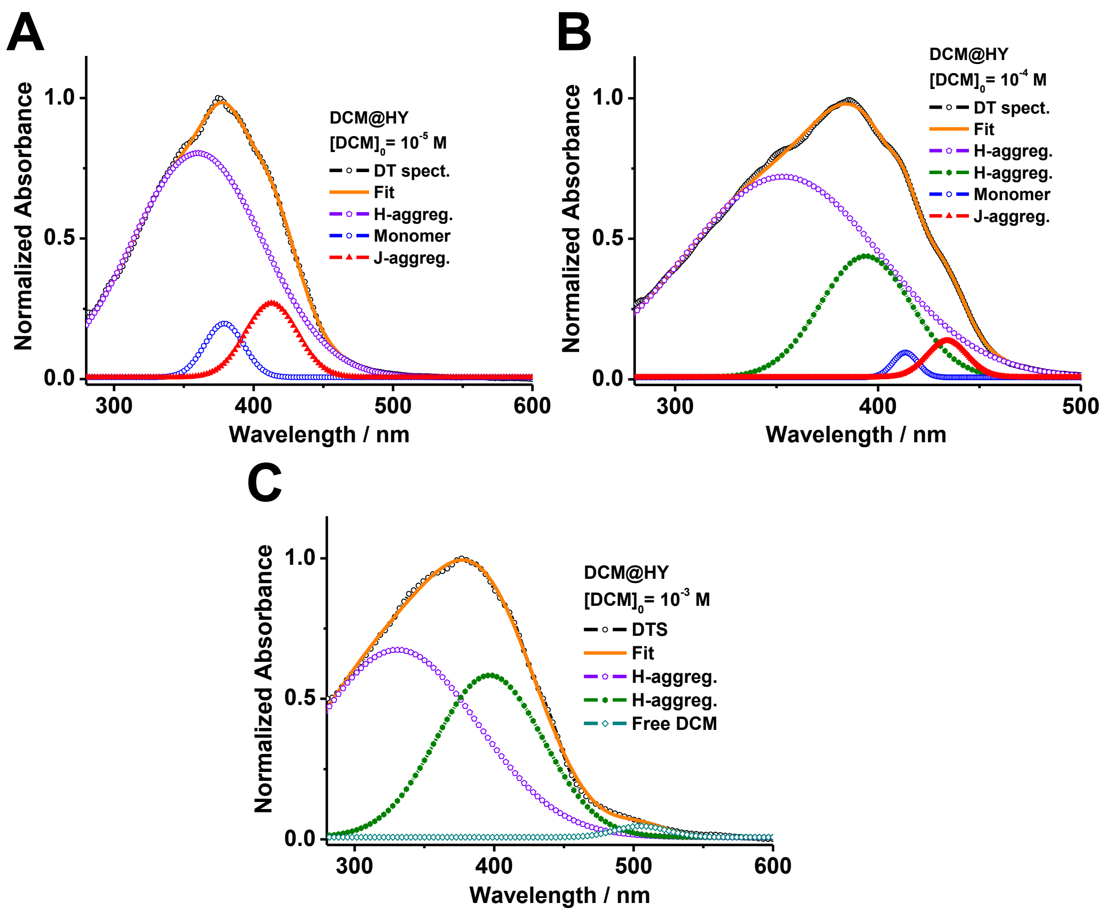

Figure S2. Deconvolution of the UV-visible DT spectra of DCM@HY in dichloromethane suspensions at different initial DCM concentrations: (A) 1 × 10^{-5} M, (B) 1 × 10^{-4} M, and (C) 1 × 10^{-3} M.

DCM@HY	H-aggregates		Monomers		J-aggregates		Free DCM	
$[DCM]_0$/M	λ_{Abs} (nm)	Area (%)	λ_{Abs} (nm)	Area (%)	λ_{Abs} (nm)	Area (%)	λ_{Abs} (nm)	Area (%)
10^{-5}HY	**360**	83	**379**	6	**413**	11	-	-
10^{-4}	**353, 394**	76, 21	**414**	1	**434**	2	-	-
10^{-3}	**331, 397**	63, 36	-	-	-	-	**505**	1

Table S1. Values of the maximum intensity wavelengths and normalized (to 100%) integral areas observed in the UV-visible DT spectra of the formed species of DCM upon interaction with HY zeolite in dichloromethane suspensions. The spectral components were obtained by a spectral deconvolution of the experimental data. The

error associated to the wavelength at the maximum absorption intensity (λ_{Abs}) in the deconvolution analysis is about ~ 5 nm.

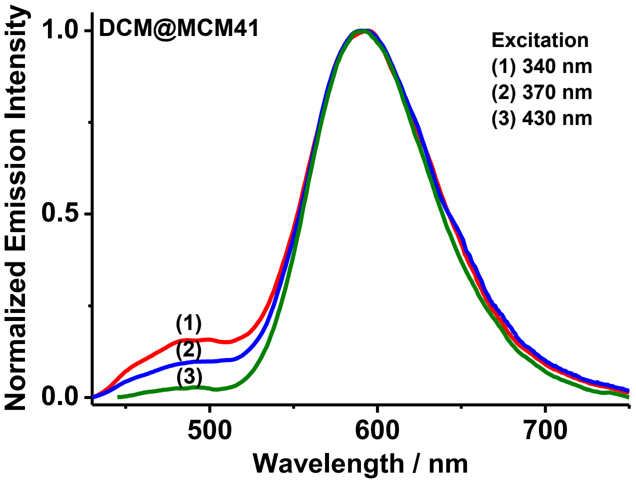

Figure S3. Normalized (to the maximum of intensity) UV-visible fluorescence spectra of DCM interacting with MCM-41 in dichloromethane suspension. The initial dye concentration is 1 × 10^{-4} M. For emission, the excitation wavelengths are indicated in the Inset.

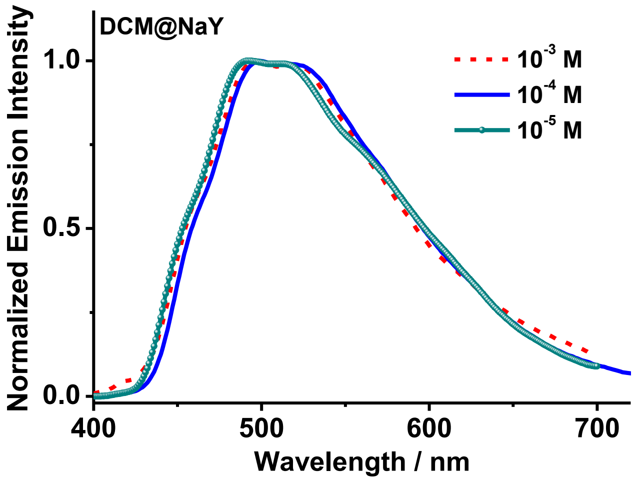

Figure S4. Normalized (to the maximum of intensity) UV-visible fluorescence (excitation wavelength = 370 nm) spectra of DCM interacting with NaY zeolite in dichloromethane suspensions at different initial dye concentrations: 1 × 10^{-3} (dashed line), 1 × 10^{-4} (solid line), and 1 × 10^{-5} (scattered-solid line) M.

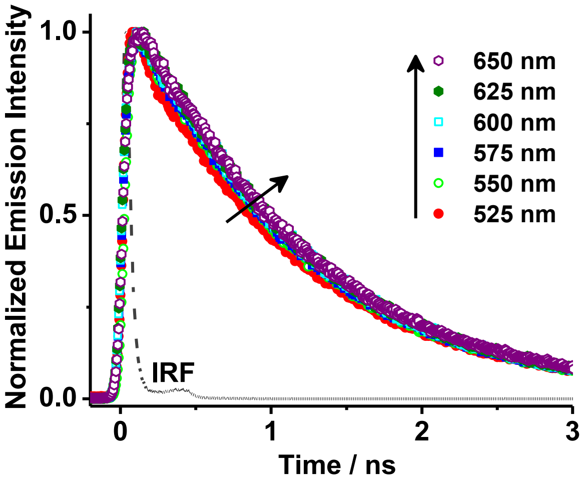

Figure S5. Normalized (to the maximum of intensity) magic-angle emission decays of DCM in dichloromethane solution upon excitation at 371 nm and observing at the wavelengths indicated in the Inset.

System	λ_{obs}/nm	τ_1/ns	a_1/%	τ_2/ns	a_2/%
**DCM/dichloromethane**	525	0.20	19	1.13	81
	550		15		85
	575		12		88
	600		11		89
	625		10		90
	650		9		91

Table S2. Values of time constants (τ_i) and normalized to (100) pre-exponential factors (a_i) obtained from the fit of the emission decays of DCM in dichloromethane solution upon excitation at 371 nm and observation as indicated in the Table.

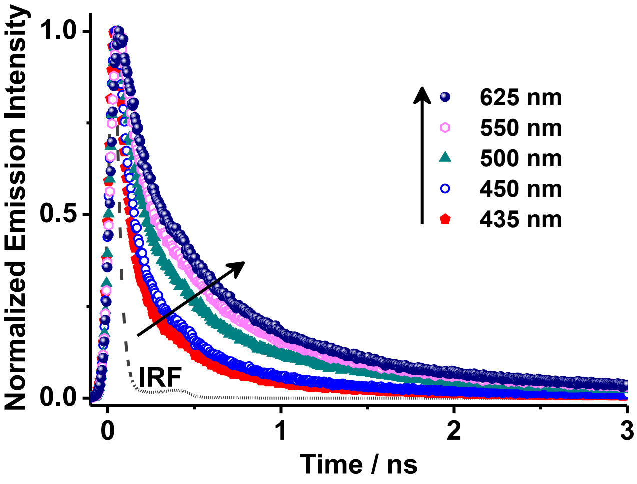

Figure S6. Normalized (to the maximum of intensity) magic-angle emission decays of DCM (1 × 10^{-3} M) interacting with HY zeolite in dichloromethane suspension. The sample was excited at 371 nm and observed at the wavelengths indicated in the Inset.

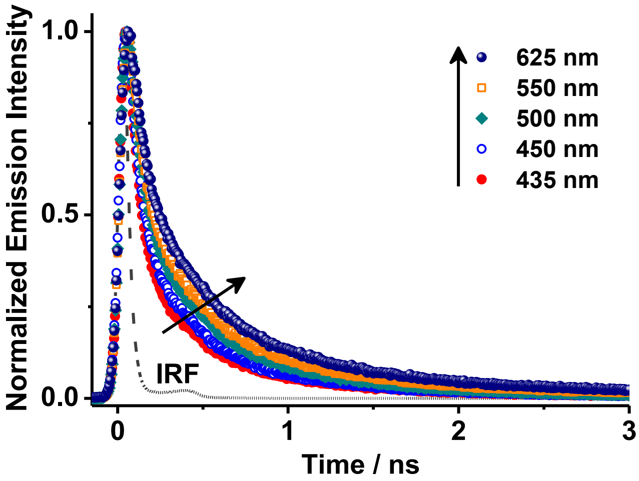

Figure S7. Normalized (to the maximum of intensity) magic-angle emission decays of DCM (1 × 10^{-4} M) interacting with HY zeolite in dichloromethane suspension. The sample was excited at 371 nm and observed at the wavelengths indicated in the Inset.

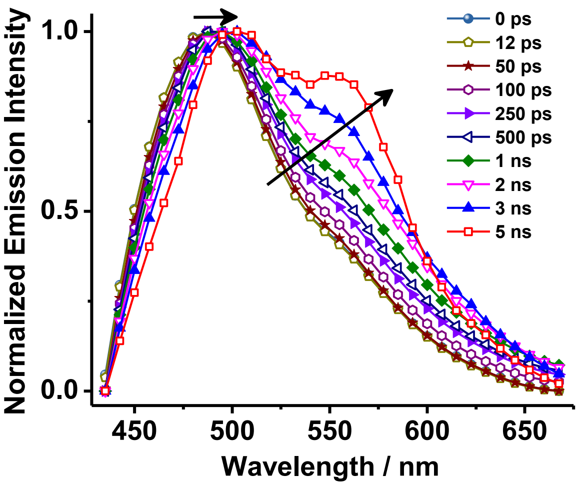

Figure S8. Normalized time-resolved emission spectra (TRES) of DCM (1 × 10^{-4} M) interacting with HY zeolite in dichloromethane suspension gated at the indicated delay times after excitation at 371 nm.

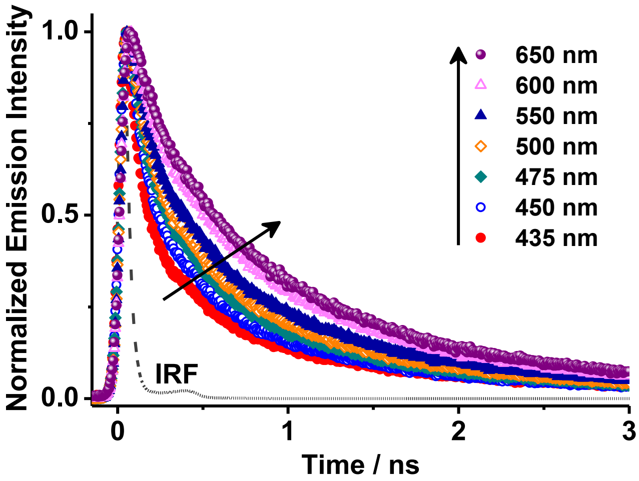

Figure S9. Normalized (to the maximum of intensity) magic-angle emission decays of DCM (1 × 10⁻⁴ M) interacting with NaX zeolite in dichloromethane suspension. The sample was excited at 371 nm and observed at the wavelengths indicated in the Inset.

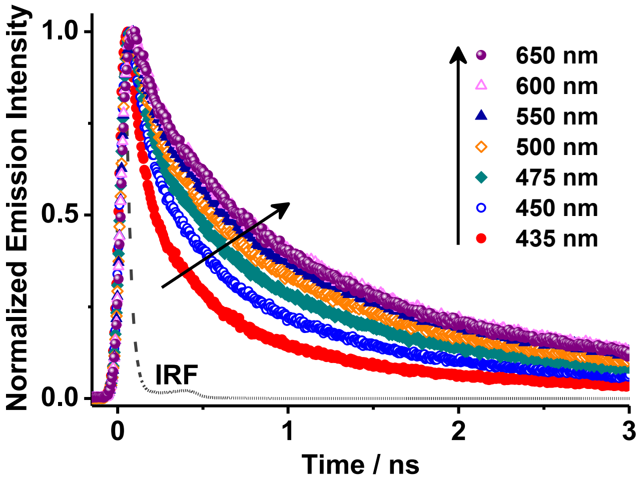

Figure S10. Normalized (to the maximum of intensity) magic-angle emission decays of DCM (1 × 10^{-4} M) interacting with NaY zeolite in dichloromethane suspension. The sample was excited at 371 nm and observed at the wavelengths indicated in the Inset.

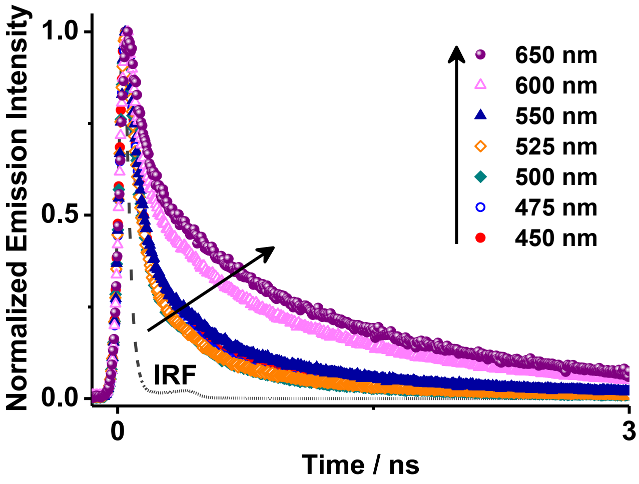

Figure S11. Normalized (to the maximum of intensity) magic-angle emission decays of DCM (1 × 10^{-4} M) interacting with MCM-41 in dichloromethane suspension. The sample was excited at 371 nm and observed at the wavelengths indicated in the Inset.

Table S3. Values of time constants (τ_i) and normalized (to 100) pre-exponential factors (a_i) obtained from the fit of the emission decays of DCM (1 × 10^{-4} M) interacting with HY, NaX, NaY, and MCM-41 in dichloromethane suspensions upon excitation at 371 nm at the observation wavelengths indicated in the Table.

		H-aggregates		J-aggregates		Monomers		Free DCM	
**Host**	**λ_{em} /nm**	**τ_1 (± 15) /ps**	**a_1 /%**	**τ_2 (± 0.07) /ns**	**a_2 /%**	**τ_3 (± 0.58) /ns**	**a_3 /%**	**τ_4 (±0.16) /ns**	**a_4 /%**
HY	435	79	78	0.36	20	3.87	2	1.10	-
	450		75		22		3		-
	500		62		26		3		9
	550		57		23		9		11
	625		53		20		10		17
NaX	435	96	70	0.40	29	2.75	1	1.10	-
	450		68		31		1		-
	475		65		34		1		-
	500		49		28		1		22
	550		43		25		2		30
	600		39		23		3		35
	650		36		20		5		39
NaY	435	99	65	0.36	31	3.30	4	1.10	-
	450		59		33		8		-
	475		55		33		12		-
	500		43		29		18		10
	550		40		28		20		12
	600		37		27		22		14
	650		35		27		22		16
MCM-41	450	65	86	0.35	13	2.46	1	1.10	-
	475		73		24		3		-
	500		61		35		3		1
	525		59		29		9		3
	550		56		26		11		7
	600		46		22		13		19
	650		41		16		15		28
